# Discontinuation of hydroxychloroquine in older patients with systemic lupus erythematosus: a multicenter retrospective study

**DOI:** 10.1186/s13075-020-02282-0

**Published:** 2020-08-17

**Authors:** Ruth Fernandez-Ruiz, Nicole Bornkamp, Mimi Y. Kim, Anca Askanase, Anna Zezon, Chung-E Tseng, H. Michael Belmont, Amit Saxena, Jane E. Salmon, Michael Lockshin, Jill P. Buyon, Peter M. Izmirly

**Affiliations:** 1 Division of Rheumatology, Department of Medicine, New York University School of Medicine, New York, NY USA; 2 Department of Population Medicine, Harvard Medical School, Boston, MA USA; 3 Division of Biostatistics, Department of Epidemiology & Population Health, Albert Einstein College of Medicine, New York, NY USA; 4 Division of Rheumatology, Department of Medicine, Columbia University College of Physicians & Surgeons, New York, NY USA; 5 Division of Rheumatology, Englewood Hospital and Medical Center, Englewood, NJ USA; 6 Division of Rheumatology, Department of Medicine, Hospital for Special Surgery, Weill Cornell Medical College, New York, NY USA

**Keywords:** Lupus, Hydroxychloroquine, Maculopathy

## Abstract

**Background:**

Although hydroxychloroquine (HCQ) is a mainstay of treatment for patients with systemic lupus erythematosus (SLE), ocular toxicity can result from accumulated exposure. As the longevity of patients with SLE improves, data are needed to balance the risk of ocular toxicity and the risk of disease flare, especially in older patients with quiescent disease. Accordingly, this study was initiated to examine the safety of HCQ withdrawal in older SLE patients.

**Methods:**

Data were obtained by retrospective chart review at three major lupus centers in New York City. Twenty-six patients who discontinued HCQ and thirty-two patients on HCQ matched for gender, race/ethnicity, and age were included in this study. The primary outcome was the occurrence of a lupus flare classified by the revised version of the Safety of Estrogens in Lupus Erythematosus: National Assessment version of the Systemic Lupus Erythematosus Disease Activity Index (SELENA-SLEDAI) Flare composite index, within 1 year of HCQ withdrawal or matched time of continuation.

**Results:**

Five patients (19.2%) in the HCQ withdrawal group compared to five (15.6%) in the HCQ continuation group experienced a flare of any severity (odds ratio [OR] = 1.28; 95% CI 0.31, 5.30; *p* = 0.73). There were no severe flares in either group. The results were similar after adjusting for length of SLE, number of American College of Rheumatology criteria, low complement levels, and SELENA-SLEDAI score, and in a propensity score analysis (OR = 1.18; 95% CI 0.23, 6.16; *p* = 0.84). The analysis of time to any flare revealed a non-significant earlier time to flare in the HCQ withdrawal group (log-rank *p* = 0.67). Most flares were in the cutaneous and musculoskeletal systems, but one patient in the continuation group developed pericarditis. The most common reason for HCQ withdrawal was retinal toxicity (42.3%), followed by patient’s preference (34.6%), other confirmed or suspected adverse effects (15.4%), ophthalmologist recommendation for macular degeneration (3.8%), and rheumatologist recommendation for quiescent SLE (3.8%).

**Conclusions:**

In this retrospective study of older stable patients with SLE on long-term HCQ, withdrawal did not significantly increase the risk of flares.

## Background

Antimalarials are among the most frequently prescribed medications in systemic lupus erythematosus (SLE). The use of antimalarials, in particular hydroxychloroquine (HCQ), has been associated with numerous benefits: reduction of flares, including during pregnancy [1, 2]; reduction of the risk of neonatal lupus [3–6]; protection against organ damage [7–9]; reduction of the risk of thrombosis [10–14]; improvement in lipid profile [13, 15, 16]; hyperglycemia [17]; bone mineral density [18]; and survival [14, 19, 20].

Despite a track record of safety, HCQ is associated with the development of maculopathy [21]. The risk of ocular toxicity was originally thought to be rare. From 1983 to 2003, prevalence estimates were 0–0.5% for patients taking ≤ 6.5 mg/kg of actual body weight/day for < 6 years and remained low for longer durations of use [22, 23]. A large longitudinal observational study of 3995 patients revealed a 1% estimated prevalence of HCQ retinopathy among those taking the drug for > 5 years, which increased with longer duration of use or an accumulated dose of > 1000 g [24]. However, as ophthalmologic techniques become more sensitive, the incidence and prevalence of maculopathy have been increasing. Based on 10-2 visual field and spectral-domain optical coherence tomography, the prevalence of HCQ maculopathy is 7.5% after at least 5 years [25]. At a daily dosage between 4.0 and 5.0 mg/kg, the prevalence of retinal toxicity remains < 2% within the first 10 years but approaches 20% after 20 years. It was reported that patients taking a mean daily dose > 5.0 mg/kg had approximately 10% risk of retinal toxicity within 10 years of treatment and almost 40% risk after 20 years [25]. In addition to maculopathy, HCQ is also rarely associated with life-threatening cardiotoxicity. A recent systematic review identified over 80 case reports and case series describing cardiomyopathy due to antimalarials, which is generally associated with high cumulative doses of HCQ or chloroquine use [26]. Cardiac events with antimalarials can range from reversible or subclinical dysfunction to severe irreversible damage requiring pacemaker use or heart transplantation [27–29].

The longer life expectancy for patients with SLE will likely result in a substantial population of older SLE patients [30–34] who may be at significant risk for HCQ adverse events, including maculopathy and cardiomyopathy. Accordingly, the objective of this study was to examine the safety of HCQ withdrawal in older SLE patients with long-standing disease.

## Methods

### Study population

SLE cases were identified from three SLE databases at New York University School of Medicine, Hospital for Special Surgery, and Columbia University College of Physicians and Surgeons. Data were obtained by retrospective chart review. This study was approved by the Institutional Review Board of New York University School of Medicine.

### Inclusion/exclusion criteria

Twenty-six patients met the following inclusion criteria: (1) ≥ 4 American College of Rheumatology (ACR) criteria for SLE [35] or Systemic Lupus International Collaborating Clinics (SLICC) classification criteria [36], (2) disease duration ≥ 5 years, (3) HCQ use of 200–400 mg/day ≥ 5 years, (4) discontinuation of HCQ at age ≥ 55 years, (5) prednisone ≤ 7.5 mg/day, and (6) a clinical Safety of Estrogens in Lupus Erythematosus: National Assessment version of the Systemic Lupus Erythematosus Disease Activity Index (SELENA-SELEDAI) [37] instrument score of ≤ 4. The comparator group comprised 32 age- (within 3 years), gender-, and race/ethnicity-matched subjects with clinical data available during the time period matching the patients who discontinued HCQ.

### Study design, outcome measures, and data collection

This was a retrospective chart review study. The primary outcome was the occurrence of a lupus flare within 1 year of HCQ withdrawal or matched time of continuation, by the revised version of the SELENA-SLEDAI Flare composite index (rSFI) that separates mild from moderate flares, evaluates each organ system separately, and incorporates increases in corticosteroid dose and/or addition of immunosuppressive agents [37, 38]. Secondary outcomes included incidence of mild, moderate, or severe flares, classified by the rSFI. The adjudication of flares and their severity was performed by two of the investigators (PI and JB), blinded to the patients’ group. Additional clinical secondary outcomes included death from any cause, venous thrombosis, and cardiovascular events within 1 year of HCQ discontinuation or matched time of continuation.

### Statistical methods and analysis

Categorical variables were summarized by computing counts and proportions of patients (%). Continuous variables are expressed as mean ± standard deviation (SD) or median with interquartile range (IQR), as appropriate. Baseline characteristics were compared between the HCQ withdrawal and continuation groups in analyses unadjusted for matching and confounders using the chi-square or Fisher’s exact test for categorical variables and the two-sample *T* test or Mann-Whitney *U* test for continuous variables. To assess the association between HCQ status and the occurrence of flare during the 12-month period of interest, which was initially considered a binary outcome, generalized linear mixed models (GLMM) using the logit link were fit to the data to account for the matched design and potential confounders. Because the sample size and number of flares in the study limited the number of confounders that could be included as independent covariates in the model, a propensity score analysis was also conducted. Specifically, for each patient, a propensity score was estimated from a logistic regression model that was fit with HCQ withdrawal status as the outcome and years since diagnosis of SLE, years of HCQ use, low C3 or C4, SLEDAI, number of ACR criteria for SLE, history of lupus nephritis, immunosuppressive use, and presence of anti-double stranded DNA antibodies as predictors. Given that the patients were already matched by age, race, and gender, the covariate adjustment method was used in the propensity analysis, where the propensity score was included as a covariate, along with HCQ withdrawal status, in the GLMM model. Missing data rates ranged from 0 to 13.7% across study variables and were addressed in the GLMM analysis using multiple imputation with chained equations. The distribution of time to flare was estimated by the Kaplan-Meier method and compared between groups using the log-rank test. Two-sided *p* values < 0.05 were considered significant for all statistical analyses. All analyses were performed using SAS version 9.4 and SPSS version 26.

## Results

### Patient demographics and disease characteristics at baseline

Fifty-eight patients were included in the study. Twenty-six patients discontinued HCQ, and 32 patients on HCQ were matched at the time of discontinuation. Baseline characteristics are summarized in Table 1. There were no significant differences between the two groups with regard to age, gender, race/ethnicity, C3 and C4 levels, clinical SLEDAI score, proportion of patients with positive anti-dsDNA antibodies, or history of lupus nephritis. The duration of SLE was longer in the HCQ withdrawal group than in the HCQ continuation group (24.3 ± 10.6 years vs. 17.8 ± 11.8 years, *p* = 0.03). Patients who discontinued HCQ had a slightly lower number of accumulated ACR classification criteria than the comparator group (4.6 ± 0.9 vs. 5.4 ± 1.5, respectively, *p* = 0.04). The proportion of patients on prednisone (7.7% vs. 15.6%) and other immunosuppressants (15.4% vs. 25%) were lower in the HCQ withdrawal group. C3 and C4 levels were marginally higher in the HCQ withdrawal group (108.1 ± 16.4 vs. 100.5 ± 27.6 for C3; 26.0 ± 10.6 vs. 21.3 ± 10.3 for C4) as compared to the comparator group, with the proportion of patients with low C3 or C4 being higher in the HCQ continuation than in the HCQ withdrawal group (14/32 [43.8%] vs. 3/25 [12%], respectively; *p* = 0.02). The proportion of patients with type 2 diabetes mellitus (T2DM) at baseline was similar between the groups (7.7% of patients in the HCQ withdrawal group vs. 6.3% in the comparator group). A higher proportion of patients in the HCQ withdrawal group was taking statins at baseline compared to the HCQ continuation group (5/26 [19.2%] vs. 3/32 [9.4%]).

**Table 1:** Baseline characteristics of the study subjects per group

	**HCQ withdrawal (*N*** **= 26)**	**HCQ continuation (*N*** **= 32)**	***p*** **value**
**Age (HCQ discontinued or matched)**	60.4 ± 4.1	59.8 ± 4.3	0.53
**Female gender**	25 (96.2%)	31 (96.9%)	1.00
**Race/ethnicity**			0.94
White	7 (26.9%)	11 (34.4%)	
Black	8 (30.8%)	9 (28.1%)	
Asian	6 (23.1%)	6 (18.8%)	
Hispanic	5 (19.2%)	6 (18.8%)	
Duration of SLE, years*	24.3 ± 10.6 (*n* = 25)	17.8 ± 11.8 (*n* = 28)	0.03
Duration of HCQ use, years**	13.0 (8–23, *n* = 23)	14.0 (6–22, *n* = 27)	0.70
Number of ACR criteria met*	4.6 ± 0.9	5.4 ± 1.5	0.04
History of arthritis	20 (76.9%)	23 (71.9%)	0.89
History of lupus nephritis	11 (42.3%)	14 (43.8%)	1.00
History of serositis	8 (30.8%)	10 (31.3%)	1.00
**Complement, C3**	108.1 ± 16.4 (*n* = 20)	100.5 ± 27.6	0.12
**Complement, C4**	26.0 ± 10.6 (*n* = 21)	21.3 ± 10.3	0.09
Low C3 or C4*	3/25 (12.0%)	14/32 (43.8%)	0.02
**Presence of anti-dsDNA Ab**	7 (28.0%, *n* = 25)	9 (29.0%, *n* = 31)	1.00
**Immunosuppressive use**	4 (15.4%)	8 (25.0%)	0.52
AZA only	0	2 (6.3%)	
MMF only	0	3 (9.4%)	
MTX only	1 (3.8%)	1 (3.1%)	
Others only^†^	2 (7.7%)	1 (3.1%)	
Combination^‡^	1 (3.8%)	1 (3.1%)	
**Prednisone use**	2 (7.7%)	5 (15.6%)	0.44
**Statin use**	5 (19.2%)	3 (9.4%)	0.45
**Clinical SELENA-SLEDAI score**	0.2 ± 0.8	0.2 ± 0.8	0.70
**SELENA-SLEDAI score**	0.9 ± 1.4	1.8 ± 1.8	0.08

Values are expressed as *n* (%) for categorical variables and mean ± SD (standard deviation) or median (interquartile range [IQR]) for continuous variables

*ACR* American College of Rheumatology, *Anti-dsDNA Ab* anti-double stranded DNA antibodies, *AZA* azathioprine, *HCQ* hydroxychloroquine, *MMF* mycophenolate mofetil, *MTX* methotrexate, *SELENA-SLEDAI* Safety of Estrogens in Lupus Erythematosus: National Assessment version of the Systemic Lupus Erythematosus Disease Activity Index, *SLICC* Systemic Lupus International Collaborating Clinics

*Statistically significant difference by *T* test or Mann-Whitney *U* test, *p* < 0.05

**Median (IQR)

^†^Any patient on a single immunosuppressant other than systemic steroids, azathioprine, mycophenolate mofetil, and methotrexate

^‡^Any patient on combination therapy, excluding antimalarials and topical or systemic steroids

### Withdrawal of HCQ does not significantly increase the risk of flares in patients over 55 with quiescent SLE

**Fig. 1:**
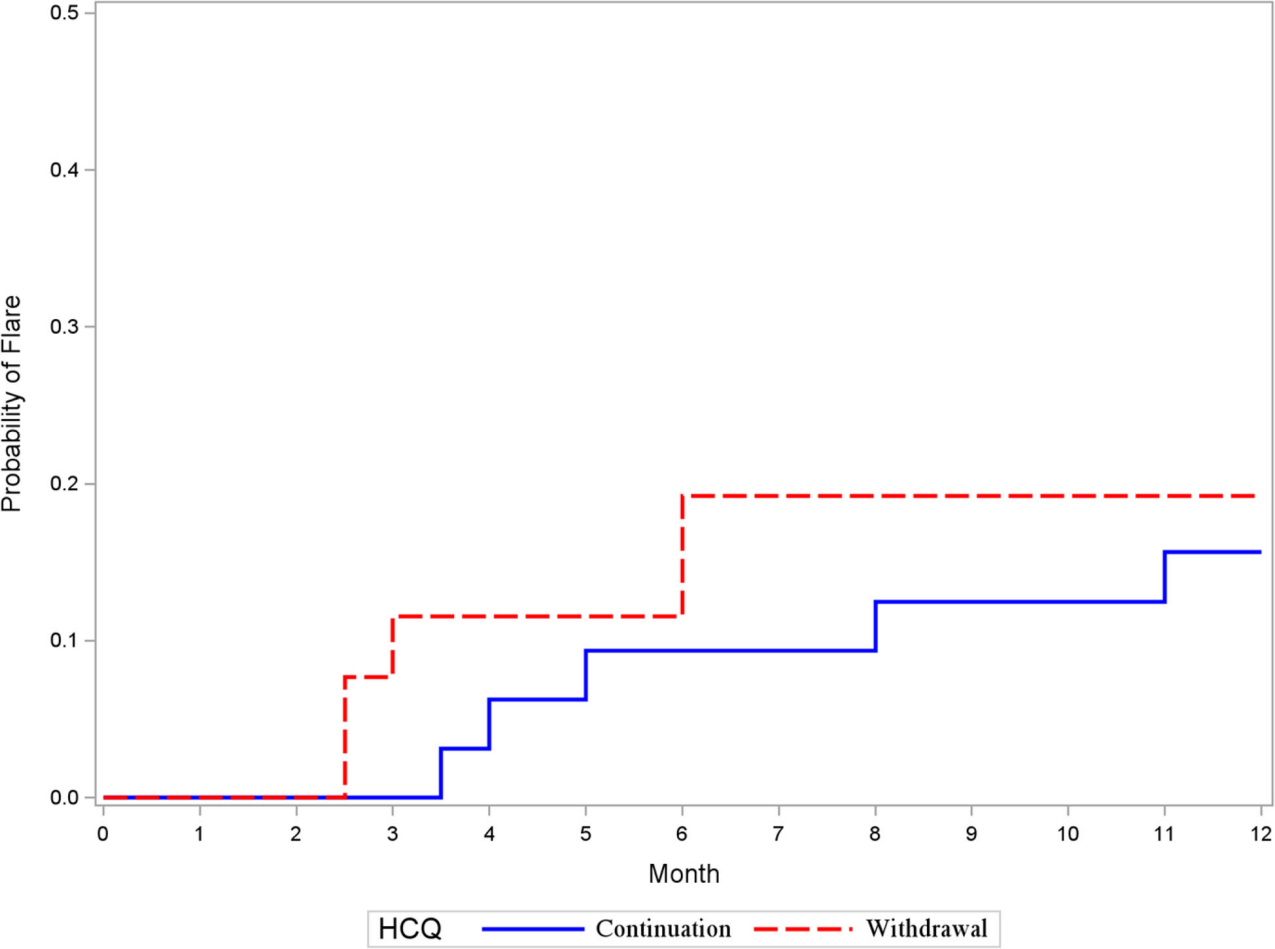
Kaplan-Meier plot of time to any flare in the HCQ withdrawal and HCQ continuation groups. SLE flares (defined by the revised version of the SELENA-SLEDAI Flare composite index) are represented by corners in the curves. The log-rank test was used to compare the curves (*p* = 0.67). HCQ, hydroxychloroquine; SELENA-SLEDAI, Safety of Estrogens in Lupus Erythematosus: National Assessment version of the Systemic Lupus Erythematosus Disease Activity Index; SLE, systemic lupus erythematosus

Five out of 26 patients in the HCQ withdrawal group (19.2%) and 5 out of 32 patients in the HCQ continuation group (15.6%) experienced a flare of any severity, corresponding to an estimated odds ratio of 1.28 (95% CI 0.31, 5.30; *p* = 0.73). After adjusting for years since diagnosis of SLE, low C3 or C4, number of ACR criteria, and SELENA-SLEDAI score (i.e., the baseline characteristics that differed between the HCQ withdrawal and continuation groups at the *p* < 0.10 level in Table 1), results were similar (OR = 1.31; 95% CI 0.18, 9.49; *p* = 0.78). The estimated odds ratio for flare from the propensity score analysis was lower (OR = 1.18; 95% CI 0.23, 6.16; *p* = 0.84). The Kaplan-Meier plots of time to flare for both the HCQ continuation and withdrawal groups are shown in Fig. 1 (log-rank = 0.67).

There were no severe flares during the 12 months following HCQ discontinuation or continuation. The rate of moderate flares was lower in the HCQ withdrawal group than in the HCQ continuation group (7.7% vs. 15.6%), corresponding to an unadjusted odds ratio of 0.45 (95% CI 0.10, 2.72; *p* = 0.37). After adjusting for propensity score, the estimated odds ratio for moderate flare was 0.50 (95% CI 0.07, 3.82; *p* = 0.50).

The clinical manifestations and treatment of flares are described in Table 2. Four patients in the HCQ withdrawal group developed cutaneous manifestations, including alopecia and pernio, discoid, or malar rash. One of these patients also developed arthritis, and another patient had polyarthritis requiring methotrexate. In the HCQ continuation group, there were three patients with cutaneous flares (discoid rash and alopecia), two of which also had polyarthritis. One additional patient who continued HCQ had polyarthritis as the only clinical manifestation, and another patient had pericarditis in this group. No patient in either group developed new manifestations of lupus during the study period.

**Table 2:** Clinical manifestations and treatment of flares according to the study group

**Patient**	**Time to flare (months)**	**Clinical manifestations**	**Flare (rSFI)**	**SELENA-SLEDAI score**	**Treatment**
**HCQ withdrawal group**					
1	2.5	Localized rash (discoid)	Mild	5	HCQ 400 mg/day
2	2.5	Localized rash (pernio) and arthritis (< 3 joints)	Mild	8	None for 1 year, followed by HCQ
3	3	Arthritis (> 3 joints)	Moderate	4	MTX 7.5–10 mg/week
14	6	Localized rash (malar) and alopecia	Mild	4	HCQ 400 mg/day
26	6	Extensive rash (discoid)	Moderate	2	HCQ 200 mg/day, followed by HCQ 400 mg/day
**HCQ continuation group**					
28	5	Serositis (pericarditis)	Moderate	4	Pred 30 mg/day + MMF increased to 2 g/day
30	11	Arthritis (> 3 joints)	Moderate	4	NSAIDs followed by Pred 20 mg/day
43	4	Alopecia and arthritis (> 3 joints)	Moderate	6	HCQ 600 mg/day
49	3.5	Extensive rash (discoid) and alopecia	Moderate	7	HCQ 600 mg/day, followed by CQ 250 mg/TIW
54	8	Extensive rash (discoid) and arthritis (> 3 joints)	Moderate	6	MTX 7.5 mg/week

CQ chloroquine, *HCQ* hydroxychloroquine, *MMF* mycophenolate mofetil, *MTX* methotrexate, *NSAID* non-steroidal anti-inflammatory drugs, *Pred* prednisone, *rSFI* revised version of the SELENA-SLEDAI Flare composite index, *SELENA-SLEDAI* Safety of Estrogens in Lupus Erythematosus: National Assessment version of the Systemic Lupus Erythematosus Disease Activity Index, *TIW* three times per week

### Reasons for HCQ discontinuation

The most common reason for HCQ discontinuation was retinal toxicity (11/26, 42.3%), followed by patient’s preference (9/26, 34.6%), other confirmed or suspected adverse effects (4/26, 15.4%), ophthalmologist recommendation for macular degeneration (1/26, 3.8%), and rheumatologist recommendation for quiescent SLE (1/26, 3.8%). One patient discontinued HCQ for biopsy-proven cardiac toxicity. No lupus flares occurred in the patients who discontinued HCQ due to maculopathy or cardiotoxicity. Two patients in the HCQ continuation group developed maculopathy after the 12-month post-matching period, requiring discontinuation of the drug. As maculopathy was diagnosed after matching and chart reviewing process, and due to a lack of follow-up after HCQ discontinuation, these patients were maintained in the HCQ continuation group.

### Metabolic, cardiovascular, and thrombotic outcomes and mortality

No patients in the HCQ withdrawal group (excluding patients with T2DM at baseline) and only one out of 30 patients in the comparator group developed T2DM during the 12 months following HCQ discontinuation or time of matching. There were no acute coronary syndromes, strokes, or other events associated with arterial thrombosis in either group during the 12-month study period. In the HCQ withdrawal group, one patient developed a postsurgical episode of unilateral lower extremity deep venous thrombosis and pulmonary embolism that was managed as a provoked venous thromboembolic event with 3 months of anticoagulation. One patient in the HCQ discontinuation group was started on a statin during the study period. There were no deaths during the 12-month post-matching period in any of the groups.

## Discussion

As the longevity of our SLE populations increases, new challenges arise for which data are sparse to non-existent. This study leverages comprehensive information abstracted from three major SLE centers led by experts in the field who maintain robust longitudinal datasets. We provide the first evidence-based guidance with regard to the consequences of HCQ withdrawal in the elderly. In our retrospective study of patients age 55 or older with quiescent SLE, the withdrawal of HCQ did not significantly increase the rate of any lupus flares when compared to a matched group of patients continuing HCQ. While 60% of the flares in the HCQ withdrawal group were mild, all flares in the comparator group were moderate. No flares occurred in patients who discontinued HCQ due to retinal or life-threatening cardiac toxicity, providing reassurance to those patients who develop antimalarial toxicity.

In considering the balance between HCQ toxicity and its benefits, addressing flares in the elderly is warranted but data to date are very limited. One study comparing 190 patients followed for 6 years during pre-menopause (mean SLE onset 27 years) to 76 patients followed for 6 years during post-menopause (mean SLE onset 52 years) revealed a decrease in activity in both groups over time, unrelated to activity at onset, menopausal status, or age at diagnosis [39]. The absence of severe flares in our study groups, both of which contained patients with long-standing SLE (mean duration of SLE 24.3 years and 17.8 years in the HCQ withdrawal and HCQ continuation groups, respectively), supports these findings.

Disease quiescence, a common situation in older lupus patients, confers more confidence in considering withdrawal of HCQ. However, results of the paradigm-changing Canadian Hydroxychloroquine Withdrawal Study provide evidence for concern [1]. Two main distinctions between our aging patients at risk for maculopathy and those reported in the Canadian study limit its applicability and merit further investigation. The average duration of HCQ use was only 3.3 ± 1.6 years in the HCQ continuation group compared to 2.8 ± 1.7 years in the placebo/withdrawal group, and the average age of the SLE patients was 45 ± 13.9 years in the HCQ continuation group compared to 44 ± 13.7 years in the placebo group. Thus, patients in the Canadian withdrawal study were younger and on HCQ for less time than the duration associated with the risk of ophthalmologic toxicity. Therefore, the conclusions of this seminal study may not be applicable to long-standing elderly lupus patients at increased risk for maculopathy.

Compounding the ocular risks of accumulated HCQ use, it has been speculated that age-related changes within the retina may potentiate susceptibility to toxic damage in elderly patients [40]. Assessment of toxicity is challenging in the elderly since the diffuse loss of fundus pigmentation with age makes bull’s-eye depigmentation harder to recognize [41]. The data suggest that elderly SLE patients indeed are those that ophthalmologists consider having “high-risk eyes” defined as HCQ use > 5 years, > 1000 g total HCQ consumption, and/or > 5 mg/kg/day HCQ daily dosing. Although no publications have identified a clear age cutoff, it has been recently suggested that patients older than 60 years old are at increased ocular risk [42]. HCQ is cleared by the kidneys and liver, and dysfunction in either organ can decrease the rate of drug removal resulting in higher blood levels [43, 44].

HCQ has been associated with multiple beneficial metabolic outcomes, including lipid-lowering effects [15, 16]. In our study, only one patient in the HCQ withdrawal group required initiation of a statin, but the specific effect of HCQ discontinuation on lipid levels could not be assessed as they were not systematically checked in these patients. Although slightly more patients in the HCQ withdrawal group were on a statin at the time of HCQ discontinuation, there was an overall low use of statins in patients in both groups, supporting previous findings that suggest low use of statins in lupus patients [45]. Statins are pleotropic agents, known to exert anti-inflammatory actions beyond their lipid-lowering effects, although their role in SLE is controversial [46–49]. The immunomodulatory effects of statins in lupus may be due to decreased expression of cell adhesion molecules, pro-inflammatory cytokines, and inhibition of type I interferon production [50, 51]. The role of statins in older lupus patients and their benefit in those unable to use HCQ due to adverse effects or toxicity should be investigated.

A protective effect of HCQ in thromboembolic events in patients with SLE was first described over three decades ago [10]; however, the doses of HCQ used by these patients were not reported and the patients described in this study were younger (mean age of 41.8 in all patients, compared to 45.5 for patients with thromboemboli) and with possibly a more active disease than patients in our study (proportion of patients on prednisone > 60%) [10]. A more recent cohort study of 232 patient with SLE also supports a protective effect of antimalarials against thrombosis (HR 0.28, 95% CI 0.08–0.90) [14], but these patients were young (mean age of 36.2 years) and the median time of HCQ use was < 5 years. Hence, the cumulative effect of HCQ and the potential for persistent antithrombotic benefits after discontinuation following long-term use remain to be elucidated. We did not identify any arterial thrombotic events, and only one venous thrombosis episode (postsurgical) occurred in the HCQ withdrawal group during the 12 months following drug discontinuation, which limits our ability to draw conclusions in regard to thrombotic outcomes in the setting of HCQ discontinuation.

Although several studies have addressed HCQ use with improved survival, most of them did not explicitly address the length of HCQ use [14]. Findings of a Latin American inception cohort study suggested a time-dependent survival benefit of HCQ, with decreased mortality in those patients who maintained HCQ use for 2 or more years [20]. In our study, all patients were on HCQ for at least 5 years before discontinuation and there were no deaths during the 12-month follow-up. However, accurate comparison of mortality rates between HCQ withdrawal or continuation groups will require longer observation times and a larger sample size.

Our study findings should be interpreted considering its limitations. First, this was a retrospective study, and most of the data were collected by chart review. The limited sample size, low number of cardiovascular and thrombotic events, and relatively short follow-up period decreased the statistical power to establish differences between the groups. We note that the minimum detectable effect size with our sample size was an absolute difference in flare rates between the HCQ withdrawal and continuation groups of 33%, assuming the rate in the latter is equal to the observed rate of 15.6%, and 80% power. In addition, subgroup analyses were not feasible due to small sample size. There are also potential biases in the selection of matching cases. Patients discontinuing HCQ by their own preference may be relatively healthier than those continuing HCQ. However, all patients included in our study had low disease activity. On the other hand, the development of maculopathy or cardiotoxicity may be indicators of previous compliance to HCQ, whereas patients who, despite reported long-term treatment with this drug, have not developed toxicity may have not been as adherent to HCQ. Since HCQ blood levels prior to discontinuation were not available for either group, we are unable to confirm this hypothesis. Finally, it is important to note that our study findings can only be applied to older (post-menopausal) lupus patients with quiescent disease, after long-standing use of HCQ. Therefore, the data should not be extrapolated to younger patients who have to discontinue HCQ because of toxicity or to older patient who are active when HCQ toxicity is identified.

Among the strengths of our study are that patients were identified from well-established multi-ethnic cohorts of lupus patients with close follow-up and strict documentation of classification criteria, disease activity, and medication use. Flares were also blindly adjudicated to limit bias. Additionally, we provide data regarding flares in older lupus patients, a demographic with limited data.

## Conclusions

In summary, despite its limitations, data from this multicenter retrospective study showed that withdrawal of HCQ in stable SLE patients older than 55 did not result in a significant increase in flares compared to matched controls. Further prospective studies will be needed to confirm these reassuring observations and to assess the potential differences in metabolic, thrombotic, and mortality outcomes from HCQ withdrawal in the elderly lupus population. Evaluation of biomarkers to identify potential predictors of flares in older lupus patients should also be considered in future studies.

## Data Availability

The data supporting the conclusions of this article are included within the article.

## Abbreviations

ACR: American College of Rheumatology
CQ: Chloroquine
HCQ: Hydroxychloroquine
IQR: Interquartile range
MMF: Mycophenolate mofetil
MTX: Methotrexate
OR: Odds ratio
rSFI: Revised version of the SELENA-SLEDAI Flare composite index
SD: Standard deviation
SELENA-SLEDAI: Safety of Estrogens in Lupus Erythematosus: National Assessment version of the Systemic Lupus Erythematosus Disease Activity Index
SLE: Systemic lupus erythematosus
SLICC: Systemic Lupus International Collaborating Clinics
T2DM: Type 2 diabetes mellitus
TIW: Three times per week
